# Association between Free Testosterone Levels and Anal Human Papillomavirus Types 16/18 Infections in a Cohort of Men Who Have Sex with Men

**DOI:** 10.1371/journal.pone.0119447

**Published:** 2015-03-20

**Authors:** Hilary K. Hsu, Todd T. Brown, Xiuhong Li, Stephen Young, Ross D. Cranston, Gypsyamber D’Souza, Lisa P. Jacobson, Otoniel Martínez-Maza, Eric C. Seaberg, Joseph B. Margolick, Frank J. Jenkins, Matthew G. Moran, Kristofer Chua, Robert K. Bolan, Roger Detels, Dorothy J. Wiley

**Affiliations:** 1 School of Nursing, University of California Los Angeles, Los Angeles, California, United States of America; 2 School of Medicine, Johns Hopkins University, Baltimore, Maryland, United States of America; 3 Bloomberg School of Public Health, Johns Hopkins University, Baltimore, Maryland, United States of America; 4 Tricore Reference Laboratories, University of New Mexico, Albuquerque, New Mexico, United States of America; 5 Department of Medicine, University of Pittsburgh, Pittsburgh, Pennsylvania, United States of America; 6 David Geffen School of Medicine at University of California Los Angeles, Los Angeles, California, United States of America; 7 Jonathan and Karin Fielding School of Public Health, University of California Los Angeles, Los Angeles, California, United States of America; 8 Desert AIDS Project, Palm Springs, California, United States of America; 9 Los Angeles LGBT Center, Jeffrey Goodman Clinic, Hollywood, California, United States of America; Penn State University School of Medicine, UNITED STATES

## Abstract

**Background:**

Human papillomavirus (HPV) types 16 and 18 cause invasive cervical cancer and most invasive anal cancers (IACs). Overall, IAC rates are highest among men who have sex with men (MSM), especially MSM with HIV infection. Testosterone is prescribed for men showing hypogonadism and HIV-related wasting. While there are direct and indirect physiological effects of testosterone in males, its role in anal HPV16/18 infections in men is unknown.

**Methods:**

Free testosterone (FT) was measured in serum from 340 Multicenter AIDS Cohort Study (MACS) participants who were tested for anal HPV16/18-DNA approximately 36 months later. The effect of log10-transformed current FT level on anal HPV16/18 prevalence was modeled using Poisson regression with robust error variance. Multivariate models controlled for other HPV types, cumulative years of exogenous testosterone use, race, age, lifetime number of receptive anal intercourse partnerships, body mass index, tobacco smoking, HIV-infection and CD4+ T-cell counts among HIV-infected, and blood draw timing.

**Results:**

Participants were, on average, 60 (+5.4) years of age, White (86%), and HIV-uninfected (56%); Twenty-four percent tested positive for anal HPV16 and/or 18-DNA (HPV16 prevalence=17.1%, HPV18=9.1%). In adjusted analysis, each half-log10 increase of FT was associated with a 1.9-fold (95% Confidence Interval: 1.11, 3.24) higher HPV16/18 prevalence. Additionally, other Group 1 high-risk HPVs were associated with a 1.56-fold (1.03, 2.37) higher HPV16/18 prevalence. Traditional risk factors for HPV16/18 infection (age, tobacco smoking; lifetime number of sexual partners, including the number of receptive anal intercourse partnerships within 24 months preceding HPV testing) were poorly correlated with one another and not statistically significantly associated with higher prevalence of HPV16/18 infection in unadjusted and adjusted analyses.

**Conclusions:**

Higher free testosterone was associated with increased HPV16/18 prevalence measured approximately three years later, independent of sexual behavior and other potential confounders. The mechanisms underlying this association remain unclear and warrant further study.

## Introduction

Gay, bisexual and other men who have sex with men (MSM) are at high risk for both human immunodeficiency virus (HIV) and human papillomavirus (HPV) infections [1–3]. Over 100 different HPVs are well characterized, of which 12–25 are sexually transmitted between partners and are associated with high risk for cancers, including HPV types 16 (HPV16) and 18 (HPV18) [4, 5]. Experts classify HPV16 and 18 together with HPV31, 33, 35, 39, 45, 51, 52, 56, 58, and 59 as strongly carcinogenic (Group 1) high-risk HPVs (hrHPVs) because of associations with cervical cancers. HPV26, 30, 34, 53, 66, 67, 68, 69, 70, 73, 82, 85, and 97 show more limited associations with human cancers and are classified as weakly carcinogenic (Group 2) hrHPVs [4]. Nearly 72–90% of HPV-associated anal malignancies and over 70% of cervical malignancies are causally related to HPV16 and 18 alone, making them of special interest [6–9]. Other genital-tropic HPVs are classified as lower risk HPVs (lrHPVs) and are associated with low-grade dysplasia including genital warts [4].

Sex hormones have been linked to HPV-related cervical cancers through the association between oral contraceptive (OC) use and parity in women [10]. For example, large human case-control studies show OC use ≥5 years increases risk for in situ and invasive cervical carcinoma by approximately 3.4-fold [11–18]. Although few studies have explored sex hormone receptor distribution in anal tissues, data suggest that androgen and estrogen receptors (AR, ER) are detectable in stratified anal epithelium, with deeper strata showing greater abundance than more superficial epithelium [19]. ERs and ARs are additionally detected in connective tissues underlying the anal epithelium [19]. Histological similarity suggest that the ectocervix and anus may both support (HPV) carcinogenesis, but no data associate HPV infections and cancers with exposures to androgens such as endogenous and exogenous testosterone, pharmacological equivalents, and their downstream metabolites.

Available data suggest that frailty, hypogonadism, and wasting disease symptoms; as well as age and obesity are associated with lower circulating serum testosterone levels [20–25]. In HIV-infected men, especially in those who develop AIDS-defining illnesses, testosterone levels are lower than in HIV-uninfected men [20, 26]. Our goal was to evaluate the association between serum free testosterone (FT) and the prevalence of anal HPV16 and/or 18 (HPV16/18) infections in a well-described cohort of MSM, and to identify possible risk factors for anal HPV infection which may affect invasive anal cancer (IAC) risk.

## Methods

### Subjects

The Multicenter AIDS Cohort Study (MACS) protocol and its substudies, including the Anal Health Study (AHS), were approved locally by institutional review boards at each of four U.S. MACS study sites: (Baltimore) Johns Hopkins Bloomberg School of Public Health, (Chicago) Northwestern University, (Los Angeles) UCLA, and (Pittsburgh) University of Pittsburgh. Written informed consent for study procedures, specimen collection and testing were provided by the sample of 340 men evaluated for this study.

Overall, the MACS has enrolled 6,972 HIV-infected and -uninfected MSM in its lifetime: 4,954 men were enrolled between 1984–1985, 668 men between 1987–1991, and 1,350 men between 2001–03 1,350 [27, 28]. By the year 2012, 2,291 MSM remained enrolled and active in the MACS, of which 49% (1131/2291) were HIV-infected, and 61% (1389/2291) had been enrolled before 2001 [1]. Physical examination data, self- and interviewer-administered questionnaires for sociodemographic, sexual and other behavior characteristics, and laboratory specimens, including blood serum and cells, are collected semi-annually.

### Sample/Specimen Selection

These analyses reflect data from two separately conducted MACS sub-studies that evaluated serum testosterone and anal HPVs among HIV-infected and -uninfected men. The testosterone study (parent study) evaluated the hypothesis that HIV-infection affected serum FT levels using a nested case-control study design. Specifically, serum specimens for 542 HIV-infected and -uninfected men ≥45 years of age were retrospectively selected from the MACS repository and tested for testosterone. Peripheral blood specimens collected from all men evaluated at each study visit were separated into cells and serum at local sites and aliquots of varying volumes were cryopreserved for long-term storage. Frozen serum specimens were shipped monthly on dry ice to the national MACS repository for all men completing a study visit. For these analyses, testosterone-tested specimens for the parent study had been frozen and thawed once, at most, prior to (testosterone) testing. Bolelli, et al. and others suggest testosterone analytes are stable in frozen serum 10 or more years [29–31] and the specimens in this study had an average interval of 3.3 years (±2.2) between collection and testing.

Case and control specimens tested for the parent study were selected from repository samples and up to six serum specimens/subject were evaluated. Controls were matched to cases based upon gender, race, enrollment period, study site, and specimen-collection (calendar) time, and age (±5 years). HIV-infected men (cases) were selected if ≥3 specimens were available, with ≥1 samples collected ≤3 years before beginning highly active antiretroviral therapy (HAART), and ≥2 samples collected within 10 years following HAART initiation. The availability and initiation of HAART varied among cases, and accordingly, temporally-matched test specimens ranged widely. For men who later enrolled in the Anal Health Study, first specimens tested for testosterone were collected between May, 1993 and February, 2006; similarly, the last specimens tested in each subject’s specimen set were collected between June 1997 and January 2010. For each man, testosterone data for only one visit were included in the analysis (as described in the “exposure of interest” section below).

HPV data were collected as part of a second sub-study that began active enrollment of 1,504 men in March 2010. Anal swab specimens were collected using a standardized clinical protocol and all active MACS men were eligible to participate [1, 32]. DNA extracted from swab specimens were tested for HPV and samples were stored for 42 days on average before HPV testing [1]; importantly, PreservCyt specimens may be stored at 2°- 30°C for up to 6 months before HPV testing (Hologic, Inc., Bedford, MA) [33].

Thus, this study is best conceptualized as a voluntary follow-up study of 340 HIV-infected and—uninfected men who were tested for FT as part of a passive, nested case-control analysis of associations between serum FT, HIV-infection and HAART (S1 Fig., S1 Table). Specifically, 340 of 542 (63%) FT-tested men from the parent study were alive and elected to undergo anal HPV testing. Non participants included 6% (33/542) of men who were lost to follow-up, 10% (54/542) of whom died before the first HPV test visit, and 21% (115/542) of whom were eligible but opted out of (HPV/cytology) testing. Together, sociodemographic, sexual and behavioral data, and the FT measurement proximal to the HPV genotyping test visit were analyzed to test the hypothesis that FT increased the probability of HPV16/18 infection.

The **outcome of interest** was HPV Type 16 or 18 DNA detected in anal swab specimens. Each specimen was collected using a standard clinical protocol: A Dacron swab was inserted approximately 2 inches beyond the anal verge, approximated to the wall and withdrawn, while rotated, over ~30 seconds before being placed into 20 mL of preservative (PreservCyt, Cytyc Corporation, Marlborough, MA) [1, 32]. A vortex mixer was used for several minutes to dislodge the collected material from swabs within the preservative containers. DNA was extracted from 250 μL of the cytology sample (Qiagen MinElute PCR Purification kit, Qiagen, Valencia, CA), 50 μL was amplified (for HPV-DNA) using the PGMY09/11 primer system [34]. Denatured PCR-products were hybridized to a probe array and were tested for 37 different HPVs types, including HPV16 and-18, 18 other Group 1 and 2, strongly and weakly carcinogenic, hrHPVs and 16 lrHPVs (Linear Array (HPV-LA) Roche Diagnostic Laboratories, Pleasanton, California) [4, 35, 36]. To evaluate contamination, positive and negative controls are run with each HPV-LA assay; data suggest the assay detects both single and multi-type infections more sensitively than earlier technologies and target amplification assays [35, 36]. Less than 1% (3/340) of first drawn cytology specimens were inadequate using HPV-LA; for those, findings from the second visit were evaluated in these analyses.

FT, the **exposure of interest,** was derived from total testosterone measured in cryopreserved serum using liquid chromatography-tandem mass spectrometry, and sex-hormone binding globulin estimated from radioimmunoassay [26]. The Vermeulen equation was used to estimate FT which closely approximates exact measures of FT obtained from equilibrium dialysis methods [37]. For this assay, the lower threshold for normal specimens drawn in the morning (AM) is 50 pg/mL [38, 39]. The FT measurement selected for these analyses was based on a focused preliminary assessment of the FT data. HAART initiation varied across the study group and the series of FT measurements for each case and matched control straddled the first visit where HAART use was reported by the index (matched) case in the parent study. Consequently, the first and last FT in the series of estimates from the parent study was collected, on average, 11.4 (+4.3) and 3.3 (+2.3) years before HPV testing, respectively. When considered together, the average slope for each subject’s FT measurements over the observation period was small, decreasing over time, and closely approximated for HIV-infected and -uninfected men: i.e., for log_10_-transformed data, β = -0.003 (+0.06) and β = -0.007 (+0.03), respectively. FT measured in serum was not statistically significantly associated with the time lapse between the last FT measurement and the first HPV test visit (for log_10_-transformed data, β = 0.014, p = 0.175). Thus, the FT measurement closest to the HPV test visit was selected and evaluated for these analyses.

### Other covariates

We evaluated demographic characteristics that were collected at semiannual visits, including age, race (White non-Hispanic vs. others); other Group 1, Group 2 and low risk HPVs; tobacco smoking, alcohol use, body mass index (kg/m^2^, BMI), HIV-infection characteristics at the HPV test visit, and Hepatitis C virus (HCV) infection, known to be associated with FT [26]. Also, men who tested positive for ≥1 of 9 other Group 1 strongly carcinogenic hrHPVs (HPV31, 33, 35, 39, 45, 51, 52, 58, or 59), for any of 9 Group 2 weakly carcinogenic hrHPVs (HPV26, 53, 66, 67, 68, 69, 70, 73, and 82), or for ≥1 of 16 lrHPVs (HPV 6, 11, 40, 42, 54, 55, 61, 62, 64, 71, 72, 81, 83, 84, IS39, or CP6108) detected by the HPV-LA assay were compared to men that tested negative, respectively [4, 35, 36]. The number of sexual partnerships and smoking and alcohol use characteristics were evaluated over three periods: (1) lifetime exposure up until the first ever MACS visit (MACS Visit 1), (2) cumulative exposure between each man’s first MACS visit and the visit 24 months before HPV testing, and (3) the last 24 months before HPV testing. Levels of alcohol and tobacco use showed no associations with HPV; thus, ever-users of alcohol and tobacco during each period were compared to non-users. Associations between cumulative pack-years of tobacco use at visit 1, between visit 1 and the FT visit, and during the last 24 months were poorly to modestly correlated (r = 0.40–0.65, p-values<0.001) and poorly associated with HPV16/18 DNA detection in exploratory univariate and multivariate analyses. However, ever having smoked prior to MACS visit 1, between the baseline MACS visit and the FT visit, and during the last 24 months of observation were strongly correlated (r = 0.64–0.97, *p*-values<0.001); for this reason, only smoking closest to the HPV test visit was included in the final model. Those with BMI ≥25 kg/m^2^ were compared to subjects with BMI <25 kg/m^2^ men. For 32 men missing BMI measures at the testosterone visit, BMI measured at the closest MACS visit was used. Men reporting >500, 201–500, and 51–200 lifetime sex partners at MACS Visit 1 were compared to men who reported ≤50 sex partners at MACS Visit 1. Men who reported ≥355, 133–354, and 63–132 partners, representing quartile cutoffs, between MACS Visit 1 and the visit 24 months before HPV testing were compared to men who reported <63 partners over the same interval. Men who reported ≥4 and 1–3 receptive anal intercourse (RAI) partners for the last 24 months prior to HPV testing were compared to those who reported none. For 5 men missing RAI partnership data for ≥1 study visits during the last 24 months of observation, the number reported at each of the last four visits with complete RAI data before HPV testing were included. Lifetime cumulative duration of exogenous testosterone use in years was calculated for men who reported ever using exogenous testosterone. For these analyses, exogenous testosterone products included Androderm (Actavis, Inc., Salt Lake City, UT), AndroGel (AbbVie Inc., North Chicago, IL), Delatestryl (SAB-Pharma Inc., Boucherville, QC, Canada), Striant (Mipharm S.p.A, Milan, Italy), Testoderm (Alza Corporation, Mountain View, CA), and Virilon (Star Pharmaceuticals, Inc., North Miami Beach, FL). Data for three MACS study sites were compared to data from the site with the largest population, and men who enrolled early in the MACS, before 2001, were compared to those enrolled in 2001–2003 to control for sociodemographic differences. Time of phlebotomy was included in the analysis to control for differences associated with diurnal variation in testosterone secretion: before vs. after noon.

### Statistical analysis

Participant characteristics were described overall, stratified by HPV16/18 infection and HIV-status, using chi-squared and Fisher’s exact test. Risk factors for HPV16/18 positivity were assessed in univariate and multivariate analysis using Poisson regression with robust error variance analyses, and prevalence ratios (PR) and 95% confidence intervals (95% CI) are reported [40]. The effect of testosterone level on HPV prevalence used half log_10_-transformed FT. Cochran-Armitage tests for trend and Pearson correlation coefficients described associations between continuous variables. Variables were eliminated from the full model using quasi-likelihood under the independence model criterion statistics over four iterations.

## Results

At the HPV study visit, on average, men were 60 (Standard Deviation (+): 5.4) years of age, White (86%, 294/340), and more than half were HIV-uninfected (56%, 189/340; Table 1). The FT measurement for the visit most closely preceding the HPV test visit varied widely (μ = 76.3 (+ 54.7) pg/mL), especially among HIV-infected men: 82.9 (+78.3) vs. 70.9 (+21.2), for HIV-uninfected men (Table 2, S2 Fig.). Only 10% (34/340) of men reported ever using exogenous testosterone and, among them, all were HIV-infected and 62% (21/34) showed BMI <25 kg/m^2^. Smoking at the first MACS visit, at the FT and the HPV test visits were correlated with one another, but not the number and character of sexual partnerships reported at these three visits or with age or detection of HPV16/18 DNA. Median FT levels between HPV16/18-infected and-uninfected men (75.2 vs. 65.5 pg/mL, *p* = 0.006) (Table 1), and HIV-infected and-uninfected men (69.1 vs. 70.2 pg/mL, *p* = 0.04) (Table 2) differed. Although 21% (72/340) of men showed clinically low FT (<50 pg/mL), HIV-infected men showed an almost 2-fold higher odds of having FT below 50 pg/mL than HIV-uninfected men in the unadjusted analysis: 28% (41/151) vs. 15% (30/189); OR = 2.04 (1.20, 3.46).

**Table 1:** Characteristics of 340 Men Who Have Sex with Men Who Were Tested for Anal HPV DNA and Free Testosterone by HPV16/18 Positivity.

Characteristic	HPV16 and/or 18^a^ Positive N (Column %)	HPV16 *and* HPV18 Negative N(Column %)	Total N(Column %)	*p* ^*b*^
**Age**				
45–49	3 (4)	6 (2)	9 (3)	NS
50–59	38 (48)	124 (48)	162 (48)	
60–69	34 (43)	121 (47)	155 (46)	
70–79	4 (5)	9 (3)	13 (4)	
80+	1 (1)	0 (0)	1 (0)	
**Race**				
White Non-Hispanic	75 (94)	219 (84)	294 (86)	0.04
Non-White	5 (6)	41 (16)	46 (14)	
**HIV Infection**				
Negative	36 (45)	153 (59)	189 (56)	0.04
Positive	44 (55)	107 (41)	151 (44)	
>500 CD4 T-cells/mm^3^	*21 (48)*	*67 (63)*	*88 (58)*	NS
≤500 CD4 T-cells/mm^3^	*23 (52)*	*40 (37)*	*63 (42)*	
**Ever smoked tobacco within last 24 months before HPV testing**				
Yes	59 (74)	186 (72)	245 (72)	NS
No	21 (26)	74 (28)	95 (28)	
**Ever drank alcohol within last 24 months before HPV testing**				
Yes	75 (94)	246 (95)	321 (94)	NS
No	5 (6)	14 (5)	19 (6)	
**HPV16/18**				
Positive	-	-	80 (24)	-
Negative	-	-	260 (76)	-
**Group 1 hrHPVs**				
HPV16, 18 only	-	-	33 (9)	NS
HPV16, 18, & other	-	-	47 (14)	
Other Group 1 hrHPVs Only (No 16/18)	-	-	105 (31)	
Negative	-	-	155 (46)	
**Group 2 hrHPVs**				
Positive	37 (46)	82 (32)	119 (21)	0.02
Negative	43 (154)	178 (68)	221 (79)	
**lrHPVs**				
Positive	54 (68)	155 (60)	209 (61)	NS
Negative	26 (32)	105 (40)	131 (39)	
**Use of exogenous testosterone**				
Yes	12 (15)	22 (8)	34 (10)	NS
No	68 (85)	238 (92)	306 (90)	
**Hepatitis C Virus** ^**c**^				
Positive	4 (5)	14 (5)	18 (5)	NS
Negative	76 (95)	246 (95)	322 (95)	
**Study Center**				
1 Baltimore	22 (28)	64 (25)	86 (24)	NS
2 Chicago	11 (14)	54 (21)	65 (19)	
3 Pittsburgh	13 (16)	39 (15)	52 (15)	
4 Los Angeles	34 (43)	103 (40)	137 (40)	
**Enrollment**				
2001–03	10 (13)	27 (10)	37 (11)	NS
Pre-2001	70 (88)	233 (90)	303 (89)	
**Number of male sex partnerships reported at baseline (MACS Visit 1)**				
Greater than 500	18 (23)	63 (24)	81 (24)	NS
201–500	21 (26)	50 (19)	71 (21)	
51–200	21 (26)	81 (31)	102 (30)	
50 or fewer	20 (25)	66 (25)	86 (25)	
**Number of male sex partners reported between MACS Visit 1 to 24 months prior to HPV testing**				
355+	19 (24)	66 (25)	85 (25)	NS
133–354	16 (20)	68 (26)	84 (25)	
63–132	25 (31)	59 (23)	84 (25)	
Fewer than 63	20 (25)	67 (26)	87 (26)	
**Number of receptive anal intercourse partners within 24 months before HPV testing**				
4+	26 (33)	69 (27)	95 (28)	NS
1–3	21 (26)	55 (21)	76 (22)	
0	33 (41)	136 (52)	169 (50)	
**BMI**				
≥25 kg/m^2^	37 (46)	152 (58)	189 (56)	NS
<25 kg/m^2^	43 (54)	108 (42)	151 (44)	
**Free Testosterone (FT) level**				
Above Median (70.1 pg/mL)	52 (65)	118 (45)	170 (50)	0.003
Median and Below	28 (35)	142 (55)	170 (50)	
	**Median (IQR)**	**Median (IQR)**	**Median (IQR)**	
**FT**	75.2 (63.0, 89.4)	65.5 (51.2, 83.3)	70.1 (53.6, 84.7)	-
**Slope of log**_**10**_ **FT Change/year**	0.000 (-0.01, 0.02)	-0.005 (-0.02, 0.01)	-0.003 (-0.01, 0.01)	NS

^a^ Positive for either HPV16 or HPV18 or both

^b^ Fisher’s Exact (2-sided) p-values: comparisons between groups in total population; NS: *p*≥0.05

^c^ HCV antibody positivity

**Table 2:** Socio-Demographic, Examination, Sexual and Other Behavioral Characteristics of 151 HIV-Infected and 189 HIV-Uninfected Men Who Have Sex with Men Who Were Tested for Anal HPV16/18 DNA and Free Testosterone at Separate Study Visits.

	HIV-infected		HIV-uninfected		Total	
	(N = 151)		(N = 189)		(N = 340)	
**Characteristics**	n (%)		n (%)		n (%)	
**HPV16/18 positive**	44 (29)		36 (19)		80 (24)	
**Use of exogenous testosterone**	34 (22.5)		0 (0)		34 (10.0)	
**Time of testosterone measurement**						
AM	44 (29)		36 (19)		80 (24)	
PM	107 (71)		153 (81)		260 (76)	
**Race**						
White Non-Hispanic	129 (85.4)		165 (87.3)		294 (87)	
Non-White	22 (14.6)		24 (12.7)		46 (13)	
**Ever smoked tobacco within last 24 months before HPV testing**						
Yes	116 (76.8)		129 (68.3)		245 (72.1)	
No	35 (23.2)		60 (31.8)		95 (27.9)	
**BMI**						
≥25kg/m^2^	69 (45.7)		120 (63.5)		189 (55.6)	
<25kg/m^2^	82 (54.3)		69 (36.5)		151 (44.4)	
	**Mean (SD)**	**Median (IQR)**	**Mean (SD)**	**Median (IQR)**	**Mean (SD)**	**Median (IQR)**
**FT Level (pg/mL)** ^a^	82.9 (78.3)	69.1 (48.0, 84.0)	70.9 (21.2)	70.2 (56.7, 83.5)	76.3 (54.7) (54.7)	70.1 (53.6, 84.7)
**Age (years)**	60.4 (5.2)	60.5 (57.6, 63.3)	59.5 (5.5)	59.3 (55.8, 63.1)	59.9 (5.4)	59.9 (56.4, 63.2)
**Number of male sexual partners reported**						
At MACS Visit 1	432 (492.1)	300 (75, 600)	294 (351.4)	101 (40, 450)	356 (424.7)	200 (50, 500)
Between MACS Visit 1–24 months before the HPV test visit	338 (710.6)	126 (59, 356)	297 (406.5)	142 (62, 351)	315 (561.7)	132 (62, 354)
**Number of receptive anal intercourse sexual partnerships reported**						
Within 24 months before HPV testing	5.4 (12.6)	1 (0, 5.0)	3.6 (8.5)	0 (0, 4.0)	4.4 (10.5)	1 (0, 4.0)
**CD4 T-cell count (T-cells/mm^**3**^)**	589 (231.7)	544 (451, 708)	914 (268.9)	888 (722, 1103)	769 (300.0)	734 (522, 969)
**Time between last FT specimen of (FT) test series and HPV testing (years)** ^b^	2.8 (1.8)	2.0 (1.0, 4.0)	3.8 (2.6)	3.0 (2.0, 5.0)	3.3 (2.3)	3.0 (2.0, 5.0)
**Time between first FT specimen of (FT) test series and HPV testing (years)**	11.1 (4.3)	13.0 (7.0, 15.0)	11.7 (4.3)	14.0 (8.0, 15.0)	11.4 (4.3)	13 (8.0, 15.0)
**Slope (change log**_**10**_ **FT/year between first to last FT visit)**	-0.003 (0.06)	-0.002 (-0.02. 0.01)	-0.007 (0.03)	-0.003 (-0.01, 0.01)	-0.006 (0.45)	-0.003 (-0.01, 0.01)

^a^ Wilcoxon two-sample test (exact test): comparing FT medians for HIV-infected vs. uninfected, two-sided test: Z = -.8128 *p* = 0.42

^b^ Wilcoxon two-sample test (exact test): comparing time between index FT and HPV tests, for HIV-infected vs. uninfected, two-sided test: Z = -3.7069, *p* = 0.0002

Nearly 24% (80/340) of participants tested HPV16/18-positive; of these, 17% (58/340) tested positive for HPV16 and 9% (31/340) showed HPV18-DNA. Only 41% (33/80) tested positive for HPV16 or-18 alone from among the 12 Group 1 hrHPVs [4]. Nearly 31% (105/340) of all men tested positive for Group 1 hrHPVs other than HPV16/18, and men who tested positive for Group 2 hrHPVs showed 1.6 times higher prevalence of HPV16/18-DNA than did men who tested negative for Group 2 hrHPVs, 31% vs. 19% (p = 0.02). The prevalence of HPV16/18 was nearly as common among men testing positive for ≥1 lrHPVs than not: 26% vs. 20% (Table 1). HIV-infected men having ≤500 CD4+ T-cells/mm^3^ had a higher prevalence of HPV16/18 infection than HIV-infected men with >500 cells/mm^3^ and HIV-uninfected men: 37% (23/63) vs. 24% (21/88) vs. 19% (36/189), respectively (Cochran-Armitage Test for Trend, *p*<0.01; Table 2). Men whose FT visit was more closely approximated to the HPV DNA test visit were no more likely to test positive for HPV16/18 than those with longer intervals, measured in years (PR = 0.99 (0.92, 1.07)), and the time interval between FT and HPV test visits was poorly correlated with FT (*r* ^2^ = 0.04, *p* = 0.40). Additionally, the mean and variation of FT levels for HPV16/18-infected men was greater than that for men that tested positive for other Group 1 hrHPVs and those who tested negative for all Group 1 viruses (S3 Fig.).

The multivariate analysis showed that the prevalence of HPV16/18-DNA detected in anal specimens increased 1.9-fold for each half log_10_ increase in FT (PR = 1.90 (95% CI: 1.11, 3.24)), even after controlling for age, race, cumulative years of exogenous testosterone use, self-reported lifetime number of sexual partnerships, BMI, tobacco smoking, HIV-infection and CD4+ T-cell count, among the HIV-infected men, and the timing of blood draws (Table 3). Non-Whites showed 2.3-fold higher prevalence of HPV16/18-DNA, though the estimate in the final model was not statistically significant (p ≥0.05) (Table 3). Prevalence of other Group 1 hrHPVs showed a 1.6-fold higher prevalence of HPV16/18 compared to those uninfected with other Group 1 viruses; however, adjusted analyses showed no statistically significant associations between HPV16/18 infection and Group 2 hrHPVs or lrHPVs (i.e., PR = 1.26 (0.81, 1.96) and 1.14 (0.74, 1.77)). Although not statistically significant, the adjusted model showed HIV-infected men with ≤500 CD4+ cells/mm^3^ had a 1.6-fold higher prevalence of HPV16/18 than did HIV-uninfected men; and, the HPV16/18 prevalence was 1.2-times higher for HIV-infected men showing >500 CD4+ cells/mm^3^. The fully-adjusted analysis suggested that the number of receptive anal intercourse partnerships within the 24 months preceding the HPV test visit did not statistically significantly affect the prevalence of HPV16/18 DNA detected in anal swab specimens (e.g., for 1–3 and ≥4 partners: PR = 1.42 (0.89, 2.28) and 1.23 (0.76, 1.98), respectively), nor did partnerships reported earlier in the study period (Table 3).

**Table 3:** Univariate and Four Iterations of Multivariate Evaluation of Risk Factors for Anal HPV16/18 DNA Positivity for 340 Men Who Have Sex with Men.^a^

				Unadjusted	Multivariate Adjusted			
					Model 1	Model 2	Model 3	Model 4
		N	HPV16/18Positive (% of total)	Prevalence Ratio (PR) (95% CI)	PR^b^ (95% CI)	PR^c^ (95% CI)	PR^d^ (95% CI)	PR^e^ (95% CI)
**Half Log**_**10**_ **Increase Testosterone (centered around the mean)**		340	80 (24)	**1.96 (1.38, 2.79)**	**1.80 (1.05, 3.11)**	**1.82 (1.03, 3.21)**	**1.87 (1.06, 3.27)**	**1.90 (1.11, 3.24)**
**Cumulative Years of (Exogenous) Testosterone Use (per year)**		340	80 (24)	1.08 (0.98, 1.20)	0.93 (0.82, 1.05)	0.94 (0.82, 1.07)	0.94 (0.82, 1.07)	0.92 (0.81, 1.05)
**Other Group 1 hrHPVs (HPV 31, 33, 35, 39, 45, 51, 52, 58, or 59)**								
Yes		152	47 (31)	**1.76 (1.19, 2.60)**	**1.71 (1.11, 2.62)**	**1.63 (1.06, 2.50)**	**1.62 (1.06, 2.47)**	**1.56 (1.03, 2.37)**
No		188	33 (18)	1	1	1	1	1
**Group2 hrHPVs (HPV 26, 53, 66, 67, 68, 69, 70, 73, or 82)**								
Yes		119	37 (31)	**1.60 (1.09 2.33)**	1.21 (0.78, 1.88)	1.26 (0.81, 1.95)	1.25 (0.81, 1.93)	1.26 (0.81, 1.96)
No		221	43 (19)	1	1			
**Low Risk HPVs (HPV 6, 11, 40, 42, 54, 55, 61, 62, 64, 71, 72, 81, 83, 84, IS39, or CP6108)**								
Yes		209	54 (26)	1.30 (0.86, 1.97)	1.28 (0.79, 2.07)	1.25 (0.78, 1.99)	1.22 (0.77, 1.94)	1.14 (0.74, 1.77)
No		131	26 (19)	1	1			
**Age**								
		340	80 (24)	1.00 (0.92, 1.08)	1.00 (0.96, 1.05)	1.00 (0.94, 1.22)	1.00 (0.93, 1.21)	0.99 (0.95, 1.23)
**Race**								
White		294	75 (26)	2.35 (1.00, 5.49)	**2.57 (1.14, 5.81)**	**2.66 (1.18, 6.00)**	**2.77 (1.19, 6.44)**	2.27 (0.97, 5.30)
Non-White		46	5 (11)	1	1	1	1	1
**HIV Infection**								
HIV-infected (CD4 T-cells/mm^3^)								
>500		88	21 (24)	1.25 (0.78, 2.01)	1.22 (0.73, 2.03)	1.15 (0.69, 1.90)	1.16 (0.70, 1.90)	1.23 (0.76, 2.00)
≤500		63	23 (37)	**1.92 (1.24, 2.97)**	**1.73 (1.01, 2.04)**	1.62 (0.97, 2.71)	1.58 (0.95, 2.64)	1.61 (0.96, 2.70)
Uninfected		189	36 (19)	1	1	1	1	1
**Time of Testosterone Measurement**								
AM		255	61 (24)	1.07 (0.68, 1.68)	1.13 (0.66, 1.92)	1.10 (0.66, 1.83)	1.09 (0.65, 1.83)	0.99 (0.62, 1.57)
PM		85	19 (22)	1	1	1	1	1
**Number of Lifetime Male Intercourse Partners at MACS Visit 1**								
500+		81	18 (22)	0.96 (0.55, 1.67)	0.94 (0.50, 1.76)	0.90 (0.48, 1.66)	0.89 (0.48, 1.67)	0.92 (0.50, 1.68)
201–500		71	21 (26)	1.27 (0.75, 2.15)	1.17 (0.66, 2.07)	1.12 (0.64, 1.96)	1.09 (0.62, 1.93)	1.14 (0.66, 1.98)
51–200		102	21 (21)	0.89 (0.52, 1.52)	0.77 (0.45, 1.30)	0.80 (0.47, 1.35)	0.79 (0.46, 1.34)	0.81 (0.48, 1.36)
≤50		86	20 (23)	1	1	1	1	1
**Sum of Number of Male Intercourse Partners Since Last Visit Reported from MACS Visit 1 to 24 Months Prior to HPV Testing**								
355+	85		19 (22)	0.97 (0.56, 1.69)	0.68 (0.34, 1.38)	0.74 (0.38, 1.44)	0.76 (0.39, 1.47)	0.66 (0.34, 1.26)
133–354	84		16 (19)	0.83 (0.46, 1.49)	0.61 (0.32, 1.14)	0.64 (0.34, 1.18)	0.66 (0.36, 1.22)	0.62 (0.34, 1.12)
63–132	84		25 (30)	1.29 (0.78, 2.15)	1.12 (0.66, 1.89)	1.15 (0.68, 1.95)	1.19 (0.70, 2.01)	1.11 (0.66, 1.87)
≤62	87		20 (23)	1	1	1	1	1
**Number of Receptive Anal Intercourse Partners in the 24 Months Prior to HPV testing**								
≥4	95		26 (27)	1.40 (0.90, 2.19)	1.08 (0.63, 1.85)	1.13 (0.67, 1.91)	1.13 (0.67, 1.91)	1.23 (0.76, 1.98)
1–3	76		21 (28)	1.42 (0.88, 2.28)	1.24 (0.74, 2.05)	1.34 (0.81, 2.20)	1.32 (0.81, 2.15)	1.42 (0.89, 2. 28)
0	169		33 (20)	1	1	1	1	1
**BMI**								
≥25kg/m^2^	189		37 (20)	0.69 (0.47, 1.01)	0.88 (0.57, 1.34)	0.86 (0.57, 1.31)	0.87 (0.57, 1.31)	0.87 (0.57, 1.32)
<25kg/m^2^	151		43 (28)	1	1	1	1	1
**Ever smoked tobacco within last 24 months before HPV testing**								
Yes	245		59 (24)	1.09 (0.70, 1.69)	0.79 (0.10, 6.25)	0.99 (0.63, 1.54)	0.99 (0.63, 1.55)	1.01 (0.65, 1.57)
No	95		21 (22)	1	1	1	1	1

^a^ Statistically significant associations appear in bold type-face, *p*<0.05

^b^ Additionally controlled for Hepatitis C virus (HCV), smoking and alcohol use prior to baseline and testosterone testing visit, cumulative pack years prior to baseline, testosterone visit, and HPV testing visit, study site, and time cohort, QIC = 508.5534.

^c^ Additionally controlled for Hepatitis C virus (HCV), alcohol use prior to HPV testing visit, study site, and time cohort, QIC = 501.2063.

^d^ Additionally controlled for study site, and time cohort, QIC = 506.7745.

^e^ QIC = 491.2723

## Discussion

HPV16/18 infections are extremely relevant in clinical practices where HIV-infected and -uninfected MSM are treated. Finding that higher serum FT, measured approximately 3 years before testing, increased risk for detecting HPV16/18-DNA in anal swab specimens suggests a causal temporal relationship. These findings support our hypothesis that testosterone, a sex steroid hormone, will affect productive HPV infections. In this study group, only HIV-infected men reported exogenous testosterone use but also showed statistically significantly lower FT levels, on average. These phenomenon are similarly reported by other investigators [20, 26], suggesting that in the absence of treatment, mean levels may have been even lower in HIV-infected than -uninfected men. Nonetheless, finding that higher FT increases risk for HPV16/18 infections some three years after its measurement may be important to carcinogenesis. HPV16/18 and 10 other hrHPVs are necessary but alone insufficient causes of human cancer [4], and persistent HPV16/18-DNA has been detected in both anal high-grade squamous intraepithelial lesions (HSIL) and IACs using worldwide samples. For example, 75–81% of HSIL and IAC lesions test positive for HPV16 DNA alone [41]. However, some might propose associations between FT and HPV16/18 infections are irrelevant because some data suggest persistent anal infections are uncommon and clearance is relatively rapid among high-risk MSM [42]. HPV-clearance may be more difficult to measure because small, persistent focal infections with low-copy number may be undetectable, even using sensitive molecular assays [5]. Nonetheless, clearance of HPV is less frequently observed for HPV16 [5, 43] than among other hrHPVs: e.g., 12.2 vs. 19.5–57.2/1000 person months in one large study [42, 44].

Most men in this study showed higher serum FT values when compared to the lower threshold for clinically normal values [38, 39]. Sex hormones have long been associated with HPV-malignancies in women [5]. For example, in vitro studies show 17β-estradiol stimulates the promoter and increases HPV oncogene expression in well-described cancer cell lines [45]. Others report estrogen and androgen receptors alike are detectable in anal squamous epithelium, making a biological association between focal anal HPV16/18 infections and FT more plausible [19]. However, we find no prior studies have evaluated the associations between FT and either HPV16/18 infections or IAC incidence. However, these data suggest that the prevalence of anal HPV16/18 infection in men nearly doubles with each half log_10_ increase of serum FT, even after controlling for the effects of other covariates, including age and exogenous testosterone use.

Like others, while some early analyses herein showed HIV-infection and lower CD4+ T-cell counts showed higher risk of HPV16/18 infection, the association was not statistically significant in the final model [46]. Nonetheless, some investigators report a positive association between CD4+ T-cell counts and infection risk at levels lower than the 500 CD4+ T-cells/mm^3^ cut-point reported here [46, 47]. Together, the direction and magnitude of our point estimates suggest that we may have lacked the power to detect a positive association between HIV and HPV16/18 infections as well as higher risk for men with lower CD4+ cell counts over men with higher values. Tobacco smoking [46, 48, 49], the number of sexual partners [32, 47, 50], and age [47, 50] have been positively associated with hrHPV infections and IACs. For example, current smokers vs. non- or never-smokers may show 1.23 to 2.59-fold higher risk for hrHPV infections and IACs [46, 48, 49]. Reports for some show that men with >100 lifetime male sex partners have a 30–40% higher risk for hrHPV seropositivity than men reporting fewer partners [47]; however, others report risk for anal hrHPVs is only elevated among HIV-uninfected MSM reporting >100 lifetime sexual partners, with no measurable effect among HIV-infected MSM [50]. Comparatively, when data described herein are stratified, we found a similar proportional distribution of men with <100, 100–500, and >500 lifetime sexual partnerships (data not shown). However, exploratory analyses still showed no statistically significant associations between the number of lifetime sexual partners and a more narrowly defined hrHPV subset, HPV16/18-DNA, either overall or among HIV-uninfected men alone. While these relationships may indicate associations between FT may more strongly influence prevalence of HPV16/18, they also suggest MACS men may have been more highly exposed to hrHPVs over their lifetimes than other MSM groups reported in the literature. Some differences may be related to differences in methodological approaches. For example, some published comparisons showing associations evaluated HPV16/18 outcomes using serology [47, 51]; however, some also reported associations between HPV16/18 and traditional risk factors such as tobacco smoking and sexual partnerships, when employing PCR-based (HPV) DNA assays similar to those reported herein [32, 46, 48–50].

HIV-infection increases the risk of hypogonadism and wasting, increasing the chances of prescribed testosterone replacement therapy [20, 23]. While as few as 15–16% of HIV-infected and -uninfected males show measurably low serum testosterone, low serum values are positively associated with higher risk of muscle wasting [20, 39, 52, 53]. Testosterone use in the absence of wasting or hypogonadism may not be recommended but would be relevant to our findings. For example, HIV-treatment guidelines suggest many MSM may be eligible for screening and treatment using testosterone: those with lower libido or erectile dysfunction, reduced bone mass or non-traumatic fractures; hot flashes, night-sweats, or fatigue and depression [54]. However, despite the relatively narrow screening and treatment recommendation, regardless of HIV-infection [39, 54], recent domestic and worldwide estimates suggest therapeutic use is increasing dramatically. For example, a 12-fold increase in testosterone prescription occurred between 2000 and 2011, with rates increasing >9-fold, from 10.3 to 98.5 monthly doses/1000 U.S. males [23, 55]. Together, these data suggest that testosterone use, therapeutically indicated or not, may be increasing and that older MSM who use testosterone may be at higher risk for HPV16/18 infections that are often linked to IACs [1, 41].

These analyses may be limited. For example, the sample size may be insufficient to detect associations between smoking, age, and sexual risk factors commonly associated with hrHPV infections in adults. Men evaluated for testosterone were unaware of their inclusion in the original investigation. Selection bias may limit our findings; for example, specimens for HIV-infected men selected from the repository for FT testing may not reflect a random sample of HIV-positive participants from the baseline cohort to which controls were matched. Further HIV-infected men surviving into the HAART era may not reflect the on-average experience or biological characteristics of the original cohort. Exogenous testosterone use, dose, route and source are not fully captured in these data, as use is recorded dichotomously at each semi-annual visit. Dichotomizing the phlebotomy time (before vs. after noon) may not adequately control for fluctuations in testosterone associated with physiologic diurnal variation. Also, other sex hormones were not measured; for example, we cannot evaluate the effect that testosterone conversion to estradiol, through the aromatase pathway, might have on HPV16/18 prevalence in these men [56]. Nonetheless, when polychotomous variables (e.g., number of sex partners) are misclassified or when misclassification varies for one variable across levels of covariates, the direction and magnitude of confounding cannot be estimated [57]. Additionally, the HPV-LA assay cannot distinguish between HPV52 and HPV33, 35, and 38, suggesting some conditions may be ambiguous; however, for these analyses, HPV33, 35, 38 and 52 are evaluated as a single categorical variable with 6 other Group 1 viruses [58]. Last, only 9 of 13 Group 2 HPVs are characterized by the HPV-LA and while the assay still reports findings for HPV-CP6108, the virus is also referred to as HPV89, a lrHPV [59].

Ideal use of testosterone-replacement therapy returns testosterone levels to age-appropriate norms, relieves symptoms, and prevents disease for deficient men without harmful side-effects [39, 60, 61]. Clinically, the results from these analyses suggest some risk may be associated with therapeutic and off-label use of testosterone in MSM who are at high risk for exposure to HPV16/18 [1, 32, 62]. A cautious approach to testosterone use in HIV-infected MSM may be indicated. Understanding associations between FT and persistent anal HPV16/18 infections will improve scientific understanding and inform therapeutic strategies.

## Supporting Information

S1 Table Disposition of 542 HIV-Infected and -Uninfected Men, Aged 45 Years and Older, Whose Specimens Were Selected from the MACS Specimen Repository for Testosterone Testing as Part of an Initial Matched-Design Longitudinal Study Evaluating Testosterone and HIV Infection; of these, 340 Men Participated in the Anal Health Study for HPV Genotyping Nearly Three Years Later. (DOCX)

S1 Fig Flow Diagram for 542 HIV-Infected and -Uninfected Men, Aged 45 Years and Older, Whose Specimens were Selected from the MACS Specimen Repository for Testosterone Testing as Part of an Initial Matched-Design Longitudinal Study Evaluating Testosterone and HIV Infection. Three Hundred and Forty Men Participated in the Anal Health Study for HPV Genotyping Nearly Three Years Later.
(TIF)

S2 Fig Two-Dimensional Plots of Free Testosterone Measurements for 340 Men Who Were Evaluated 3 Years before HPV Genotyping, Stratified by HIV-Serostatus and HPV Types 16/18 Infection Positivity. (TIF)

S3 Fig Frequency of Free Testosterone Measurements for 340 Men Who Tested Positive for HPV16/18-DNA and for Other Group 1, Strongly Carcinogenic hrHPVs, Versus Those that Tested hrHPV-Group 1-DNA Negative. (TIF)
