# Supplementary material for: Association between Free Testosterone Levels and Anal Human Papillomavirus Types 16/18 Infections in a Cohort of Men Who Have Sex with Men
Source: PLoS One. 2015 Mar 20;10(3):e0119447. doi: 10.1371/journal.pone.0119447 (PMC4368778; doi:10.1371/journal.pone.0119447)
Supplement: S1 Table — (DOCX) [file pone.0119447.s001.docx]

S1 Table: Disposition of 542 HIV-Infected and –Uninfected Men, Aged 45 Years and Older, Whose Specimens Were Selected from the MACS Specimen Repository for Testosterone Testing as Part of an Initial Matched-Design Longitudinal Study Evaluating Testosterone and HIV Infection; of these, 340 Men Participated in the Anal Health Study for HPV Genotyping Nearly Three Years Later.

|  |  | Tested for Testosterone | | | |
| --- | --- | --- | --- | --- | --- |
|  | CD4 T-Cell Count at the Testosterone Visit  (CD4 T-cells/mm^3^) | Died or Lost to Examination Follow-up before 10/01/2010  (row %) | Did Not Participate in Anal Health Study  (row %) | Participated in Anal Health Study (row %) | Total |
| HIV-Uninfected |  | 26 (9) | 56 (21) | 189 (70) | 271 |
| HIV-Infected |  | 61 (22) | 59 (22) | 151 (56) | 271 |
|  | >500 | 18 (14) | 24 (18) | 88 (68) | 130 |
|  | 350-500 | 5 (8) | 13 (20) | 47 (72) | 65 |
|  | <350 | 38 (50) | 22 (29) | 16 (21) | 76 |
