# Supplementary figures and images for: Association between Free Testosterone Levels and Anal Human Papillomavirus Types 16/18 Infections in a Cohort of Men Who Have Sex with Men
Source: PLoS One. 2015 Mar 20;10(3):e0119447. doi: 10.1371/journal.pone.0119447 (PMC4368778; doi:10.1371/journal.pone.0119447)

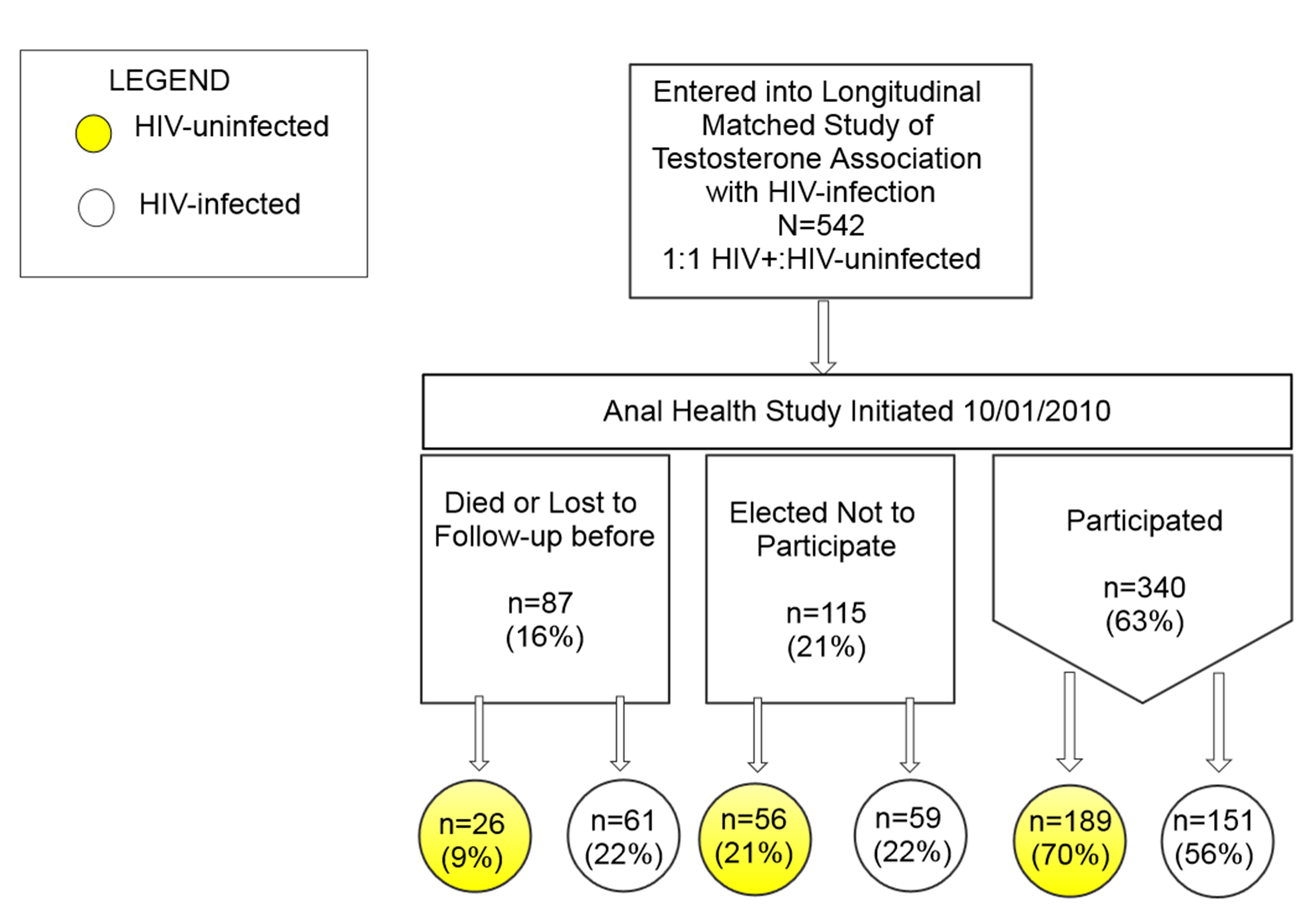

Supplement: S1 Fig — Three Hundred and Forty Men Participated in the Anal Health Study for HPV Genotyping Nearly Three Years Later. (TIF) [file pone.0119447.s002.tif]

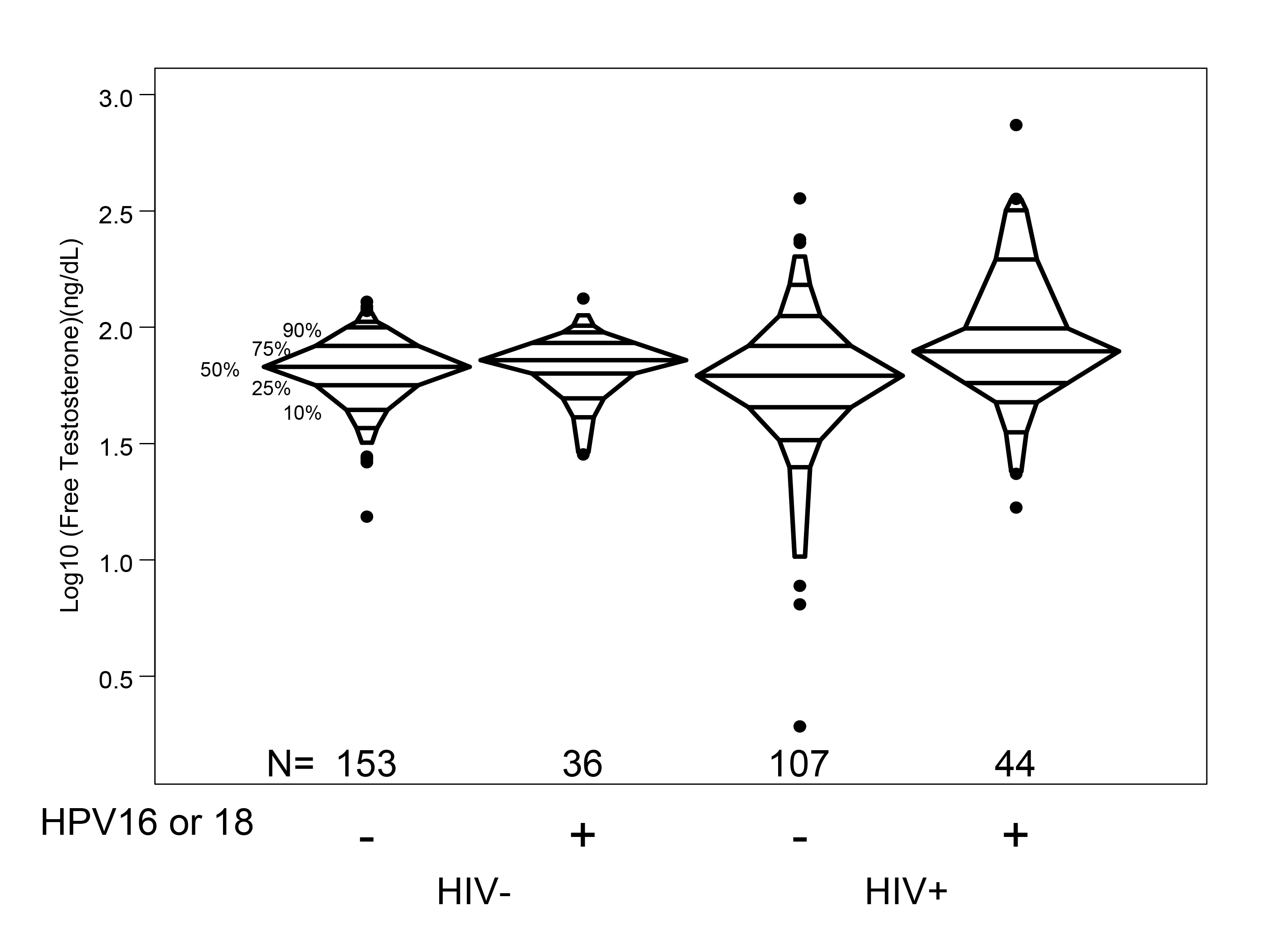

Supplement: S2 Fig — (TIF) [file pone.0119447.s003.tif]

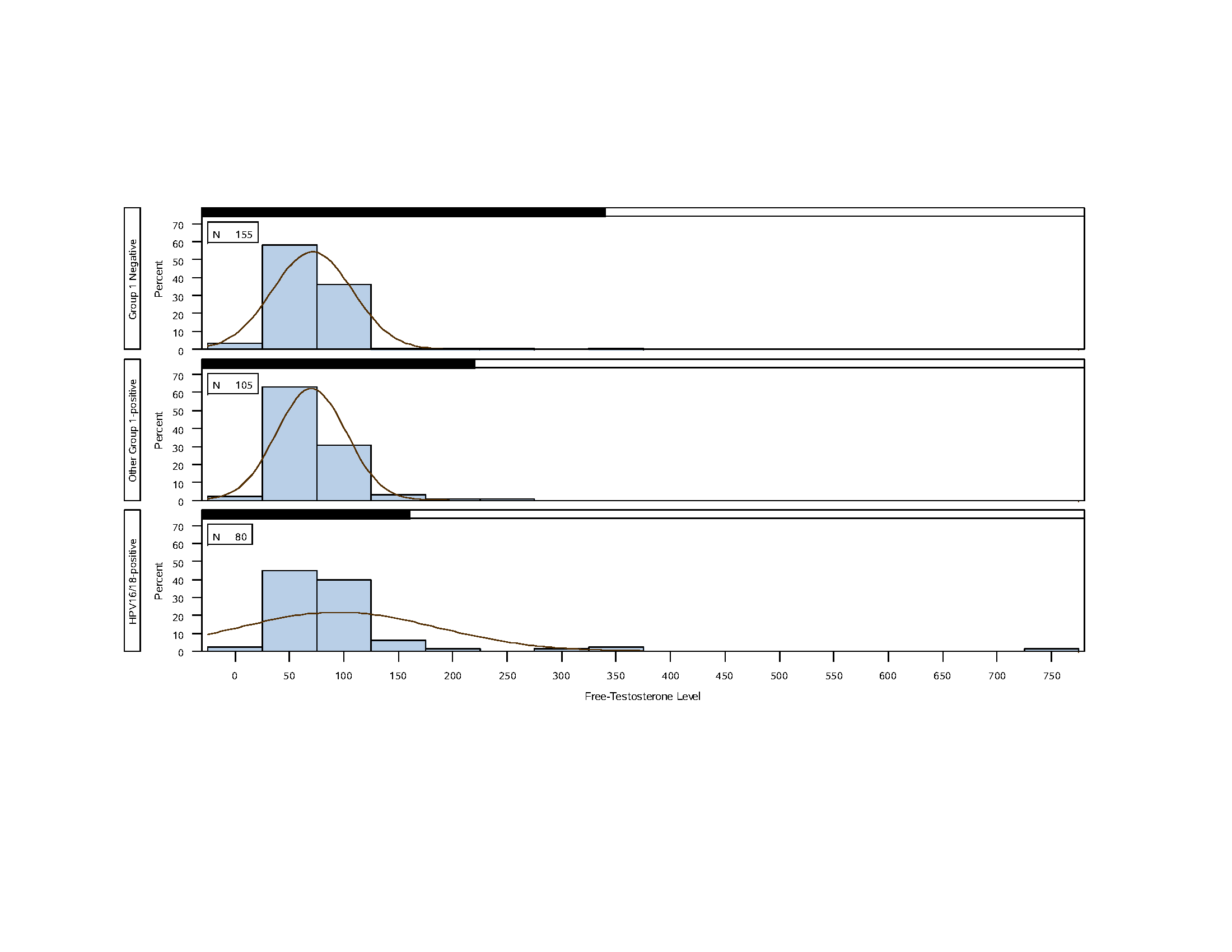

Supplement: S3 Fig — (TIF) [file pone.0119447.s004.tif]
